# An Unsupervised Learning Technique to Optimize Radio Maps for Indoor Localization

**DOI:** 10.3390/s19040752

**Published:** 2019-02-13

**Authors:** Jens Trogh, Wout Joseph, Luc Martens, David Plets

**Affiliations:** Department of Information Technology, IMEC—Ghent University, Ghent 9052, Belgium; wout.joseph@ugent.be (W.J.); luc1.martens@ugent.be (L.M.); david.plets@ugent.be (D.P.)

**Keywords:** localization, radio map, unsupervised learning, tracking, positioning, rss, fingerprinting, indoor environment

## Abstract

A major burden of signal strength-based fingerprinting for indoor positioning is the generation and maintenance of a radio map, also known as a fingerprint database. Model-based radio maps are generated much faster than measurement-based radio maps but are generally not accurate enough. This work proposes a method to automatically construct and optimize a model-based radio map. The method is based on unsupervised learning, where random walks, for which the ground truth locations are unknown, serve as input for the optimization, along with a floor plan and a location tracking algorithm. No measurement campaign or site survey, which are labor-intensive and time-consuming, or inertial sensor measurements, which are often not available and consume additional power, are needed for this approach. Experiments in a large office building, covering over 1100 m^2^, resulted in median accuracies of up to 2.07 m, or a relative improvement of 28.6% with only 15 min of unlabeled training data.

## 1. Introduction

Localization and tracking in indoor environments is important for a wide range of location-aware applications, e.g., museum guidance, navigation in a shopping mall, finding your car in a parking garage, or asset tracking in the industrial sector. Most positioning systems in GPS-denied environments rely on signal strength measurements from existing wireless network infrastructures due to their simplicity and availability, e.g., WiFi, ZigBee, or Bluetooth Low Energy (BLE) compatible devices. These Received Signal Strength (RSS) measurements can be translated to a location by making use of a path loss model and the well-known multilateration method [1]. Alternatively, with a fingerprinting technique, the position of an unknown user or object is estimated by finding the closest match in a fingerprint database (online phase) [2,3]. The fingerprint database, or radio map, is a signal space that links RSS values to positions in a building. This database is constructed in an offline phase by making use of a radio channel simulator or an elaborate measurement campaign, also known as war-driving [4]. The first approach is simulation-based, hence much faster but will generally lead to less accurate location estimations. The second approach consists of manually performing RSS measurements at known locations (grid points) and needs to be redone each time the wireless network or even the office layout undergoes changes. Other localization systems use special-purpose hardware and infrastructure like ultra-wideband (UWB), radio-frequency identification (RFID) or acoustic ranging [5,6,7]. These systems can be very accurate but the initial deployment cost will generally be much higher. Another technique is pedestrian dead-reckoning which uses inertial sensors that are typically embedded in smartphones, such as accelerometers, magnetometers, and gyroscopes [8]. The positions are calculated based on a previous position and the estimated movement of a user, by detecting steps, estimating stride lengths and the direction of motion. These systems are typically prone to drift, i.e., the positioning error accumulates over time because of noise in the inertial sensor measurements. Given the widespread use of WiFi access points or BLE beacons for indoor localization purposes, it is paramount to find methods to obtain reliable fingerprinting maps with a minimal effort. The main novel contribution of this paper is an unsupervised learning method to automatically construct, maintain, and optimize fingerprint databases, without the need for inertial sensor units, calibration, or extensive measurements. To the best of our knowledge, this is the first unsupervised learning approach that relies solely on floor plan information and unlabeled training data, i.e., signal strength measurements for which the ground truth locations are unknown. The method is experimentally validated for different path loss models and access point configurations.

## 2. Related Work

In the past, other techniques for indoor localization without the need for pre-deployment efforts, such as site surveys, measurement campaigns, or device calibrations, have been proposed [9,10,11,12,13]. A related topic is simultaneous localization and mapping (SLAM), which constructs or updates a map of an unknown environment while keeping track of the agent’s location within it [14]. This map refers to the actual physical environment, whereas this work focuses on optimizing the radio map or signal space of a building. The EZ algorithm is a configuration-free indoor localization scheme that uses a genetic algorithm and occasionally available GPS locks, e.g., at the entrance or near a window, to localize mobile devices [9]. Another technique that bypasses war-driving is UnLoc [10], which uses dead-reckoning, urban sensing, and WiFi-based partitioning. A dead-reckoning scheme is used to track a user’s smartphone between so called internal landmarks of a building, such as a distinct pattern on a smartphone’s accelerometer or an unusual magnetic fluctuation in a specific spot.

In [11], WiFi and inertial sensor information are combined with constraints imposed by a map of the indoor space of interest and augmented particle filtering is used to estimate the position concurrently with other variables such as the stride length. The RCILS algorithm is a crowdsourcing-based indoor localization system to automatically construct a radio map [15]. The trajectories of the crowdsourced data are reconstructed based on activity detection, pedestrian dead-reckoning, and a semantic graph of the floor plan. A geomagnetism-aided indoor radio-map construction method based on crowdsourcing is presented in [16]. This method utilizes magnetism sequence similarity and a clustering algorithm to form the pathway graph of a floor plan, without needing an exact floor layout, but with the assumption of straight corridors. In [17], the inertial-based readings of a smart phone for pedestrian dead-reckoning and a factor graph optimization method are combined to generate a WiFi radio map. The factor graph optimization method is used to re-estimate the trajectory by adding constraints originated from collected WiFi fingerprints and landmark positions. A joint indoor localization and radio map construction scheme is presented in [12]. This scheme transfers a source data set to a limited number of calibration fingerprints using manifold alignment. A crowdsourcing-based scheme to construct a probabilistic radio map based on parametric fitting is presented in [13]. This technique describes location signatures by transforming RSS into signal envelopes but relies on an additional localization mechanism and a very large amount of RSS samples.

Our approach does not rely on any manual calibrations, measurement campaigns [4], GPS fixes [9], landmark positions [17], or inertial sensor units [11], such as accelerometers, gyroscopes, or magnetic compasses [16], to perform pedestrian dead-reckoning [8,10,15]. Only unlabeled training data (random walks in a building) and a floor plan are needed to construct, maintain, and optimize radio maps for indoor localization, e.g., to make model-based databases more accurate or to automatically cope with changes in an office layout.

## 3. Methodology

**Figure 1:**
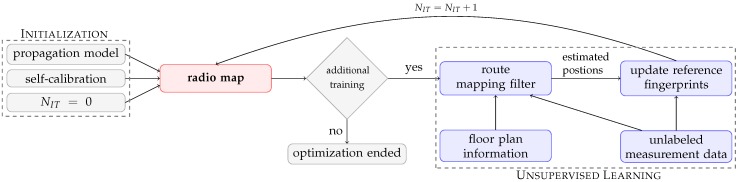
Flow graph of the proposed technique to construct and optimize a model-based radio map. $N_{IT}$ is the current training iteration.

Our approach consists of an initial radio map based on a theoretical path loss model and a self-calibration technique to match a user’s device with this radio map. The unsupervised learning technique to optimize the radio map uses a route mapping filter to reconstruct the most likely trajectory of unlabeled training data by including floor plan information and the current radio map. Next, the estimated positions from the reconstructed trajectories are used to update the reference fingerprints from the radio map. The optimization ends when the maximum number of training iterations is reached or when the learned values remain the same between two training iterations. Figure 1 shows a flow graph of the proposed technique to construct and optimize a radio map.

The proposed unsupervised learning technique could run at a central location if the network infrastructure collects all RSS measurements or locally at a user’s device if the latter collects all RSS measurements by scanning the access points in the area. The next sections describe each part in more detail, including a motivation for the chosen approach based on experiments conducted in an office building. Furthermore, three path loss models and three access point configurations are considered in the simulations and experimental validation for the proposed technique.

### 3.1. Experimental Configuration

**Figure 2:**
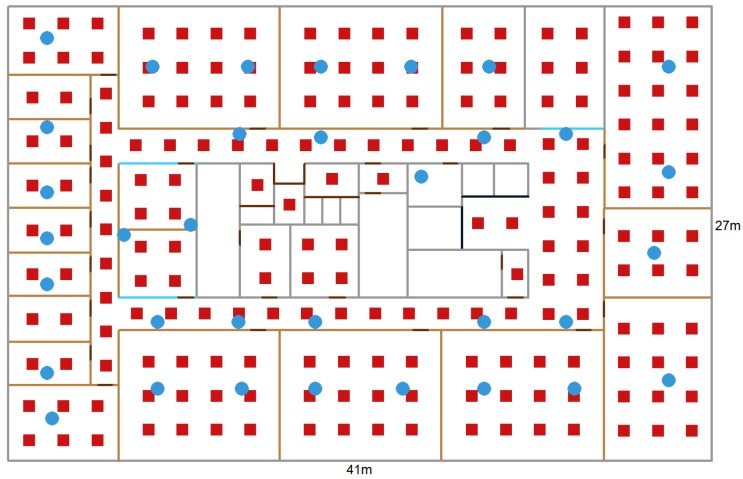
Floorplan of the office building with indication of walls, doors, elevators, access points (blue dots), and static validation locations (red squares) where a mobile node broadcasted packets at 5 Hz.

The experiments are conducted on a wireless testbed, located on the ninth floor of an office building in Ghent, Belgium, covering over 1100 m^2^ (41 m by 27 m) and is visualized in Figure 2. The inner structure of the building is made of thick concrete walls (gray) and the meeting rooms, offices, and kitchen have plaster walls (amber), wooden doors (brown), and some glass walls (blue).

**Figure 3:**
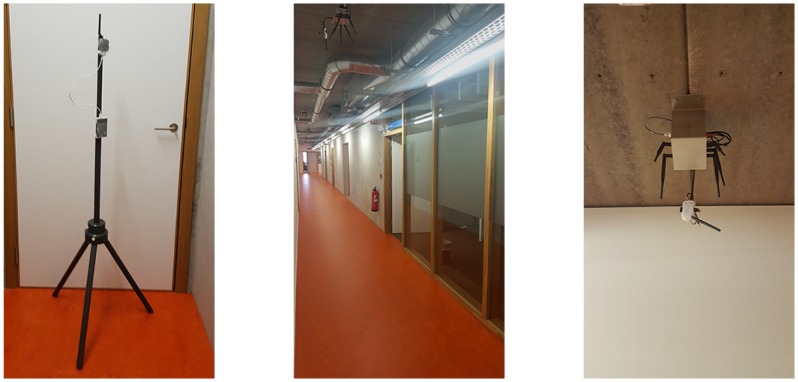
Tripod with battery powered mobile node and ceiling mounted fixed access points in corridor.

The wireless network consists of 35 fixed access points (sensor nodes) that are installed at a height of 3 m and are indicated with a blue dot. These sensor nodes are based on the Zolertia RE-mote platform, which is based on the Texas Instruments CC2538 ARM Cortex-M3 system on chip, with an on-board 2.4 GHz IEEE 802.15.4 RF interface. This interface runs at up to 32 MHz with 512 KB of programmable flash and 32 KB of RAM, bundled with a Texas Instruments CC1200 868/915 MHz RF transceiver to allow dual band operation [18]. The battery powered mobile node is based on the same platform and is mounted on a tripod with a height of 1.5 m to collect static validation data (Figure 3).

### 3.2. Radio Map

The initial fingerprint database is a simulated radio map, that can be based on any propagation model. Note that to simulate path losses, the access point locations need to be known in advance. This is independent of the proposed unsupervised learning technique because the latter solely needs an initial radio map as starting point, which can be both measurement or simulation-based. Three different path loss models are considered as initial radio map to be optimized by the self-learning technique:

- Free-space model [19]: the free-space path loss (*FSPL*) is the attenuation of radio energy between a sender and receiver antenna in idealized conditions, i.e., the antenna polarizations are perfectly matched, the environment is unobstructed free-space and the antennas are in each others far-field. The *FSPL* is calculated as follows:
  (1) $$FSPL=20\log_{10}\left(d\right)+20\log_{10}\left(f\right)-27.55$$
  FSPL [dB] denotes the free-space path loss, *d* [m] is the distance between the sender and receiver antenna and *f* [MHz] is the operating frequency, if this is set to 2400 MHz, then the model reduces to:
  (2) $$FSPL=40.05+20\log_{10}\left(d\right)$$
- IEEE 802.11 TGn model [20]: the IEEE 802.11 TGn model is a two-slope path loss model, which is suitable for path-loss predictions in office environments. The TGn is calculated as follows:
  (3) $$TGNL=\left\{\begin{array}{cc} PL_{0}+10n_{1}\log_{10}\left(d\right) & d\leq d_{br}\\ PL_{0}+10n_{2}\log_{10}\left(d\right)-32 & d>d_{br} \end{array}\right.$$
  TGNL [dB] denotes the path loss predicted by the TGn model, $PL_{0}$ [dB] is the reference path loss and is equal to 40.05 dB, n1 and n2 are the path loss exponents for the first and second part of the two-slope model and are equal to 2 and 5.2, and $d_{br}$ [m] is the breakpoint distance and is equal to 10 m. For $d\leq d_{br}$, the TGn model equals to the free-space model.
- WHIPP model [21]: the WHIPP model is a theoretical model for indoor environments that includes wall and interaction losses. This model does not use a ray tracing algorithm, but is based on a heuristic algorithm where the dominant path is searched, i.e., the path along which the path loss is the lowest. Here, the path loss values are modeled as:
  (4) $$WL=\underset{\mathrm{distance}\;\mathrm{loss}}{\underset{︸}{PL_{0}+10\gamma \log_{10}\left(\frac{d}{d_{0}}\right)}}+\underset{\mathrm{cumulated}\;\mathrm{wall}\;\mathrm{loss}}{\underset{︸}{\sum_{i}L_{W_{i}}}}+\underset{\mathrm{interaction}\;\mathrm{loss}}{\underset{︸}{\sum_{j}L_{B_{j}}}}+X_{\sigma }$$
  WL [dB] denotes the path loss predicted by the WHIPP path loss model, $PL_{0}$ [dB] is the path loss at a reference distance $d_{0}$ [m], γ [-] is the path loss exponent, *d* [m] is the distance along the path between transmitter and receiver. These two terms represent the path loss due to the traveled distance. The cumulated wall loss represents the sum of all wall losses $L_{W_{i}}$ when a signal propagates through a wall $W_{i}$. The interaction loss represents the cumulated losses $L_{B_{j}}$ caused by all propagation direction changes $B_{j}$ along the path between sender and receiver, and $X_{\sigma }$ [dB] is a log-normally distributed variable with zero mean and standard deviation σ, corresponding to the large-scale shadow fading.

### 3.3. Self-Calibration

Off-the-shelf devices are usually not capable of measuring a path loss but instead report a received signal strength (RSS) value. To be compatible with one of the theoretical path loss values, the RSS values are converted to a path loss value (or vice versa):

(5) $$\mathrm{pathloss}=-\mathrm{RSS}+RSS_{bias}$$

The RSS value is preceded by a minus sign because a higher path loss corresponds to a lower RSS value and the other way around. The $RSS_{bias}$ is a fixed offset that is calculated once and depends on the access points and the user’s device, e.g., transmitting power and antenna configuration, both of which are often unknown. Therefore, a self-calibration method [22] is used to obtain a good mapping between the measured RSS values and the reference path loss values from the radio map, also called fingerprints. This method relates the histogram of the reference radio map to the RSS histogram of a user’s device, which requires no user intervention or ground truth location data, and results in the following bias:

(6) $$RSS_{bias}=Md\left(F_{ref}^{-1}\left(y\right)-F_{meas}^{-1}\left(y\right)\right),\;y\in \left\{0.1,0.2,\ldots ,0.9\right\}$$

$RSS_{bias}$ is the estimated bias between the measurements of the user device and the fingerprint database, and is equal for all grid points and access points. The latter is only valid if all access point have the same configuration, otherwise the self-calibration could be done for each access point individually. Md(.) indicates the median value, $F_{ref}$ is the cumulative distribution functions (CDF) of the model-based reference fingerprints and $F_{meas}$ is the empirical CDF of the RSS measurements from the user’s device, multiplied by −1 to be compatible with the path loss values (Equation (5)). The assumption behind this approach is that the empirical CDF of raw measurement values, collected during a random walk, resembles the respective empirical CDF of the mean measurement values collected with the same device at several uniformly distributed known positions. For our unlabeled training data, the value of $RSS_{bias}$ stabilized after 14 s of measurement data (the sending rate was fixed at 5 Hz). Note that after the self-calibration the free-space and TGn path loss model can be seen as an intercept-fitted one-slope and two-slope path loss model.

## 4. Unsupervised Learning

This section starts with a motivation for the proposed learning technique. Next, the route mapping filter and update step for the reference fingerprints in the radio map are discussed (blue blocks in the flow graph of Figure 1).

### 4.1. Motivation

**Figure 4:**
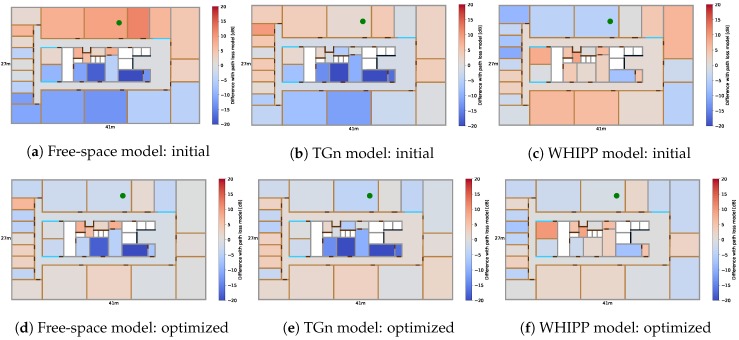
Average difference between model-based radio map and measurements, grouped per room, for one access point (green dot), before (**a**–**c**) and after the unsupervised learning (**d**–**f**).

An experiment in an indoor environment showed that measurements of neighboring locations are similar and deviations from the initial model-based radio map tend to be correlated per room and access point. A mobile node, placed on 200 separate locations, precisely measured with a laser meter, broadcasts packets at 5 Hz for 1 min while 35 fixed access points were listening. The 200 locations, ordered in a grid with a spacing of 2 m, are indicated in Figure 2 and referred to as *grid points* in the remainder of this section. Next, the median self-calibrated values of the received measurements are compared to the corresponding reference values in the model-based radio map and are grouped per room and access point. These differences are visualized for one access point in Figure 4, for each of the propagation models from Section 3.2.

The access point is indicated with a green dot and the color in each room indicates the average difference from the path loss model (after the conversion from RSS to path loss). A blue color means that the theoretical model predicts a lower path loss value than what was actually measured, i.e., the received signal is weaker than what the theoretical path loss model predicts. This is clearly visible for the free-space and TGn model in the rooms on the bottom left (Figure 4a,b). The access point is located at the top middle and the inner structure of the building is made of thick concrete walls, which weaken the radio signals significantly but the free-space and TGn model do not explicitly take into account the presence of these walls. A red color means that the theoretical model predicts a higher path loss value than what was actually measured, i.e., the received signal is stronger than what the theoretical path loss model estimates. This is the case for the WHIPP model, the same rooms on the bottom left have a reddish color because the model predicts a slightly higher path loss value than what was actually measured (Figure 4c).

Note that because of the self-calibration from Section 3.3, measurements can be stronger (lower path loss) than what the theoretical models predict because the calibration shifts all reference values with one fixed value to minimize the average differences between the measurements and the theoretical predicted values. Without this calibration phase, all deviations from the free-space path loss model would have a bluish color because the free-space model is more or less a lower limit for the measured path loss. The other access points show similar behavior but will have different values because this depends on the building’s layout and the relative location between room and access point. Comparison with the optimized radio map is discussed in Section 6 (Figure 4d–f).

Additional sources of deviation from the theoretical predicted path loss values are temporal fading and human body shadowing [23]. Temporal fading is the variability of received power over time at a static location in the propagation environment. The influence of temporal fading is diminished by taking the median value over 300 values (1 min broadcast at 5 Hz) but will have an influence if only one sample is available, e.g., when a user is tracked while walking through a building. Human body shadowing is caused by the presence of a user, who can block the line-of-sight (LoS) between a body-worn tag and a receiving node, and causes additional propagation losses [24]. The amount of additional path loss depends on both the orientation of a person and the relative placement of the mobile tag on the body. Methods to compensate for this loss include techniques that generate orientation-independent fingerprints by measuring RSS values for multiple orientations [25] or by modeling the signal attenuation caused by the human body [26]. Alternatively, the accuracy of a tracking algorithm can be improved by eliminating the shadowing caused by the human body [23]. This method estimates the orientation of a person and uses a human body compensation method in combination with the relative placement of the body-worn tag. The latter needs to be known in advance or estimated as well.

In this work, the influence of human body shadowing is eliminated by using a tripod instead of body-worn or hand-held device. Note that this is only possible to collect the static validation data, the dynamic training data will be collected by a user with a hand-held device (Section 6). The deviations are classified in three categories:

- **overall deviation**: the overall deviation represents the variation for the whole building and is used as an indication of radio map quality. A value of zero would mean that the measured path losses are exactly equal to the theoretically predicted values at all locations, for all access points.
  (7) $$RSS_{diff}^{i,j}=RSS_{meas,sc}^{i,j}-RSS_{ref,pl}^{i,j}$$
  (8) $$\mu_{diff}^{i}=\frac{1}{N_{GP}}\sum_{j}^{N_{GP}}RSS_{diff}^{i,j}$$
  (9) $$dev_{overall}=\sqrt{\frac{1}{N_{AP}\cdot N_{GP}}\sum_{i}^{N_{AP}}\sum_{j}^{N_{GP}}{\left(RSS_{diff}^{i,j}-\mu_{diff}^{i}\right)}^{2}}$$
  $RSS_{diff}^{i,j}$ is the difference between the self-calibrated measurement $RSS_{meas,sc}^{i,j}$ and the reference value $RSS_{ref,pl}^{i,j}$ for access point *i*, grid point *j*, and path loss model pl. The average difference for access point *i* is denoted by $\mu_{diff}^{i}$, the total number of grid points by $N_{GP}$, the overall deviation by $dev_{overall}$, and the total number of access points by $N_{AP}$.
- **room deviation**: the room deviation models the difference between the radio map and the measurements, averaged over a whole room.
  (10) $$DIFF_{room}^{i,k}=\frac{1}{N_{GP}^{k}}\sum_{j}^{N_{GP}^{k}}RSS_{diff}^{i,j}$$
  (11) $$\mu_{room}^{i}=\frac{1}{N_{rooms}}\sum_{k}^{N_{rooms}}DIFF_{room}^{i,k}$$
  (12) $$dev_{room}=\sqrt{\frac{1}{N_{AP}\cdot N_{rooms}}\sum_{i}^{N_{AP}}\sum_{k}^{N_{rooms}}{\left(DIFF_{room}^{i,k}-\mu_{room}^{i}\right)}^{2}}$$
  $DIFF_{room}^{i,k}$ is the average difference for access point *i* and room *k*, these values are visualized for one access point in Figure 4. $N_{GP}^{k}$ are the grid points within room *k*, $RSS_{diff}^{i,j}$ is defined in Equation (7), $\mu_{room}^{i}$ is the average room difference for access point *i*, $N_{rooms}$ are the number of rooms in the building, $dev_{room}$ is the room deviation, and $N_{AP}$ the number of access points.
- **local deviation**: the local deviation represents the variation within a room on top of the room deviation, i.e., the differences between measured path loss values and the theoretical path loss values from the radio map are similar within a room but not exactly the same for all locations in this room.
  (13) $$DIFF_{local}^{i,j}=RSS_{meas,sc}^{i,j}-RSS_{ref,pl}^{i,j}-DIFF_{room}^{i,k_{j}}$$
  (14) $$\mu_{local}^{i}=\frac{1}{N_{GP}}\sum_{j}^{N_{GP}}DIFF_{local}^{i,j}$$
  (15) $$dev_{local}=\sqrt{\frac{1}{N_{AP}\cdot N_{GP}}\sum_{i}^{N_{AP}}\sum_{j}^{N_{GP}}{\left(DIFF_{local}^{i,j}-\mu_{local}^{i}\right)}^{2}}$$
  $DIFF_{local}^{i,j}$ represents the local difference for access point *i* and grid point *j*, $DIFF_{room}^{i,k_{j}}$ is the room difference for access point *i* and room $k_{j}$, i.e., the room of grid point *j* (Equation (10)), $\mu_{local}^{i}$ is the average local difference for access point *i*, $N_{GP}$ is the number of grid points, $dev_{local}$ is the local deviation, and $N_{AP}$ is the number of access points.

Table 1 summarizes the statistics for the differences between measurements and the theoretical path loss models for all access points after self-calibration.

The differences compared to the theoretical path loss values vary from −30.3 dB to +19.7 dB for the free-space model, from −37.5 dB to +19.1 dB for the IEEE 802.11 TGn model and from −22.0 dB to +24.0 dB for the WHIPP path loss model. The average differences are around 0 due to the self-calibration phase (−0.4 dB, −0.7 dB and 0.8 dB, respectively). The minimum and maximum difference could solely be caused by outliers, a better indication of the radio map quality is the overall deviation $dev_{overall}$ (Equation (9)). This value is 10.9 dB for the free-space path loss model, 9.6 dB for the IEEE 802.11 TGn model, and 7.6 dB for the WHIPP path loss model, which is to be expected given the increased complexity. The room deviations $dev_{room}$ (Equation (12)) are 9.7 dB, 8.8 dB, and 5.7 dB, for the three path loss models, respectively. The local deviations $dev_{local}$ (Equation (15)) are similar for all three path loss models (3.5 dB, 3.5 dB and 3.7 dB, respectively).

Under the assumption that a user’s trajectory can be roughly reconstructed without knowing the ground truth locations, the $DIFF_{room}^{i,k}$ values (Equation (10)) can be learned, resulting in a radio map or fingerprint database that matches the actual measurements more closely. Consequently, this optimized fingerprint database can increase the positioning accuracy of the trajectories or static locations of other users or objects. Note that a lower $dev_{overall}$ indicates that the theoretically predicted values are a closer match to the real measurements, which makes it easier to learn the $DIFF_{room}^{i,k}$ values because mapping unlabeled training data to the correct room will be more likely.

### 4.2. Route Mapping Filter

In our approach, the trajectories of unlabeled training data are first reconstructed with a route mapping filter and subsequently passed to the learning algorithm to optimize the radio map. The route mapping filter is based on the Viterbi path [27], a technique related to hidden Markov models. It uses a motion model and floor plan information to determine the most likely path, i.e., sequence of locations, instead of only the most likely current position. These constraints ensure that no unrealistically large distances are traveled within a given time frame and no walls are crossed. By processing all available data at once, previously estimated locations can be corrected by future measurements (similar to backward belief propagation). This route mapping filter makes it possible to optimize the radio map because the estimated positions, along the reconstructed trajectories, are generally assigned to the correct room. Hence, the discrepancies between reference fingerprints and real measurements can be learned, which improves the radio map quality and positioning accuracy. This is less likely with stateless positioning techniques, where consecutive estimated positions can fluctuate between different rooms because of measurement noise and outliers. Alternatively, a Kalman filter [28] or Particle filter [29] could be used to reconstruct the trajectories but the proposed route mapping filter resulted in greater improvements and a better location accuracy.

### 4.3. Radio Map Update Step

After all training data is processed by the route mapping filter, the measurements are linked to a grid point and a room based on the estimated positions. The differences between self-calibrated RSS measurements and the corresponding reference fingerprints are grouped and averaged per room and access point. Next, the reference fingerprints in the radio map are updated and the training iteration number $N_{it}$ is increased by one (feedback loop in the flow graph of Figure 1).

(16) $$RSS_{ref,pl}^{i,j}=RSS_{ref,pl}^{i,j}+DIFF_{room}^{i,k_{j}}$$

$RSS_{ref,pl}^{i,j}$ is the reference value of access point *i*, grid point *j*, and path loss model pl, that is updated in this training iteration. $DIFF_{room}^{i,k_{j}}$ represents the average difference between a set of self-calibrated measurements from access point *i* and room $k_{j}$, i.e., the room of grid point *j*. This is similar to Equation (10) but the input locations are now based on the training data and route mapping filter instead of the 200 static validation points. It’s recommended to update the $RSS_{ref,pl}^{i,j}$ values after all training data has been processed at once. This gives the route mapping filter a chance to correct previously estimated positions by taking into account future measurements and to reduce the effect of outliers.

The update process is applied iteratively on the same unlabeled data until the learned values stagnate or when the maximum number of training iterations is reached. The reason for this is that the estimated trajectories from the unlabeled data tend to become more accurate in the next training iteration because it uses the current optimized radio map, which in turn results in a better update of the reference fingerprints. Also, if new unlabeled data becomes available, the optimization can start again to update the current radio map.

## 5. Simulation

A simulation with three different access point configurations is carried out to test the proposed unsupervised learning technique.

### 5.1. Settings

**Figure 5:**
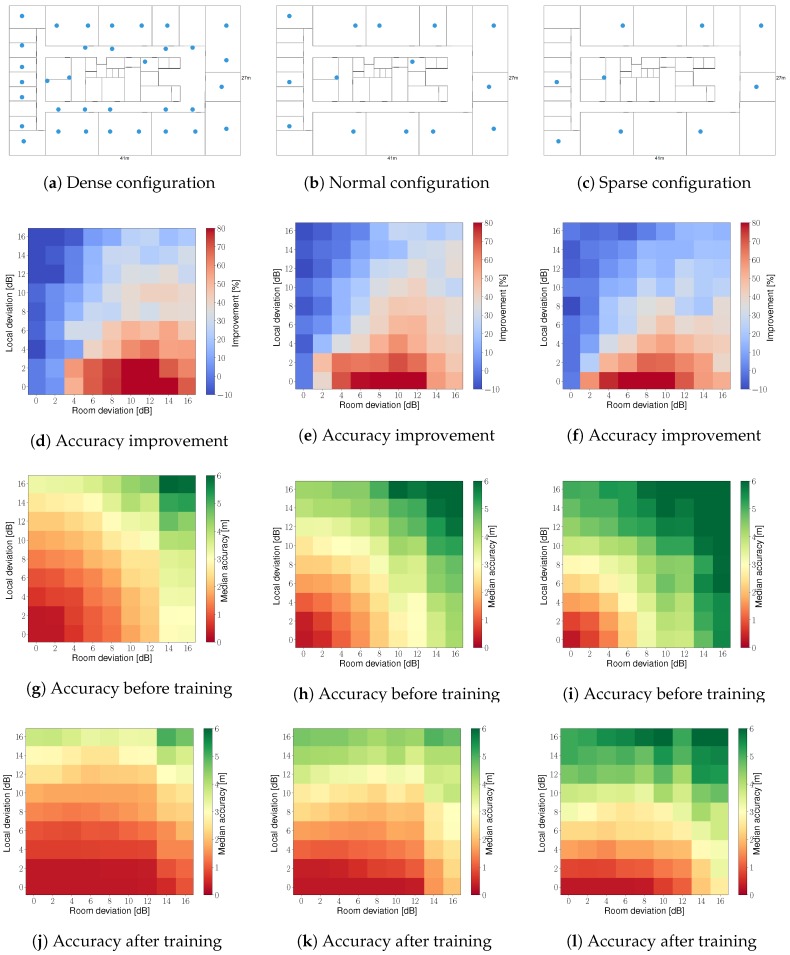
Location of the access points for the simulated scenarios with 35, 15, and 9 fixed access points: dense (**a**), normal (**b**), and sparse (**c**) configuration. The relative improvement in median accuracy after unsupervised learning with 1 h of unlabeled training data, evaluated on 1000 uniformly spread locations for the three scenario’s (**d**–**f**). The median accuracy before (**g**–**i**) and after learning (**j**–**l**) for the three scenario’s. The room deviation (*x*-axis) and local deviation (*y*-axis) vary from 0 dB to 16 dB in steps of 2 dB.

The simulation environment is the same as in Section 3.1, i.e., an office building in Ghent, Belgium, covering over 1100 m^2^. The access point configurations are subsets of the access points from Figure 2: a dense scenario with 35 access points (Figure 5a), a normal scenario with 15 access points (Figure 5b), and a sparse scenario with 9 access points (Figure 5c). The WHIPP path loss model serves as basis to simulate real measurements because this model resembles a real-world scenario more closely [21], as shown in Section 4.1.

The first simulation takes into account room and local differences: $DIFF_{room}^{i,k}$ and $DIFF_{local}^{i,j}$. Both are modeled by Gaussian noise with zero mean and are added to the reference fingerprints, i.e., the theoretical path loss values from the WHIPP model. The standard deviation is varied from 0 dB to 16 dB in steps of 2 dB, which gives a total of 81 combinations to be simulated. The test environment consists of 40 rooms and 35 access points, resulting in 1400 $DIFF_{room}^{i,k}$ values for the dense scenario. The $DIFF_{local}^{i,j}$ values are generated for each grid and access point. A grid size of 50 cm results in 4386 grid points and 153,510 $DIFF_{local}^{i,j}$ values for the dense scenario.

The second simulation considers two potential sources of additional noise on the measurements: temporal fading and human body shadowing. Both will have a major influence on the performance of the proposed unsupervised learning technique because it affects the accuracy of the reconstructed training data with the route mapping filter, as well as the learned values in the radio map update step. Both can be added together and are simulated by a single Gaussian noise with zero mean and the standard deviation is varied from 0 dB to 20 dB in steps of 2 dB. These noise values are generated each time the users passes at a location, whereas the $DIFF_{local}^{i,j}$ and $DIFF_{room}^{i,k}$ are fixed for each location and room in the building.

The objective is to learn the $DIFF_{room}^{i,k}$ values based on unlabeled training data to improve the quality of a radio map and, hence, improve the accuracy of an application that relies on this fingerprint database, such as a location or tracking system. All other values act as additional noise on the measurements that make it harder to learn the $DIFF_{room}^{i,k}$ values (which will also be the case for a real-world scenario). Also, not all $DIFF_{room}^{i,k}$ values are equally important, e.g., a correctly learned value for a large conference room will have a greater impact on the accuracy than a wrongly learned value of a small storage room. Since more random training data will pass through a larger room, it is also more likely that these values will be learned correctly.

One could argue that it is better to learn the difference for each grid point and access point individually instead of a global value per room and access point. However, simulations showed that this is unfeasible because a few learned values in a room can attract all future measurements of training data in that room, which will worsen the radio map and localization accuracy. For example, suppose training data passes at a few grid points in a room and the reference fingerprints of these grid points are updated while all other fingerprints in that room remain unchanged. Next, if new training data becomes available in that room, it will be mapped to the few updated grid points if these are closer in signal space than the actual locations, i.e., when the difference with respect to their reference fingerprints is smaller than for the actual locations. This causes the optimization technique to assign all updates to only a few grid points per room.

The training data consists of a random walk which simulates a user that walks freely in the building during 1 h with a random and variable walking speed between 4 km/h and 8 km/h. Every second, measurements are simulated by adding the generated noise values for that location and access point to the reference fingerprints. Depending on the sparse, normal, or dense scenario this number of reference fingerprints will vary between 9, 15, and 35, respectively. The quality of the radio map can be evaluated by comparing the learned values to the initially generated $DIFF_{room}^{i,k}$ values. Another indication of radio map quality is the accuracy with a static location algorithm, e.g., by taking the closest match in the optimized radio map as location estimation. This closest match is based on weighted least squares, i.e., strong signals have a greater weight than the weaker signals.

(17) $$loc_{est}=\underset{j\in GP}{\mathrm{argmin}}\sqrt{\sum_{i}^{N_{AP}}RSS_{meas,sc}^{i,j}\cdot {\left(RSS_{meas,sc}^{i,j}-RSS_{ref,pl}^{i,j}\right)}^{2}}$$

$loc_{est}$ is the estimated location, i.e., the grid point with the closest match in signal space, GP is the set of all grid points in the building, $N_{AP}$ is the set of all access points *i* that have measurements for this location update, $RSS_{meas,sc}^{i,j}$ is the self-calibrated measurement and $RSS_{ref,pl}^{i,j}$ is the reference value for access point *i*, grid point *j*, and path loss model pl. The maximum number of training iterations during the unsupervised learning is set to 10, i.e., the same unlabeled training data is used 10 times to learn the $DIFF_{room}^{i,k}$ values and update the radio map. The optimization is stopped early to speed up the process when the learned values remain the same between two training iterations (*additional training* block in the flow graph of Figure 1). Afterwards, the positioning accuracy is calculated based on 1000 uniformly spread locations with the original and optimized radio map to quantify the improvement.

### 5.2. Results

#### 5.2.1. Influence of Room and Local Deviation

The relative improvement in median accuracy as well as the median accuracy before and after the last training iteration are visualized per access point configuration in Figure 5. The *x*-axis and *y*-axis represent the room and local deviations, and the color indicates the relative improvement and median accuracy for the 81 scenarios. Each scenario, i.e., a colored square in Figure 5, is trained with 1 h of unlabeled training data, for a maximum of 10 training iterations, and is validated on 1000 uniformly spread locations. The relative improvements are similar for all three access point scenarios and the absolute accuracies are higher for the dense access point scenario, as is expected.

The highest improvement is 89.7%, the median accuracy goes from 3.3 m to 0.3 m in the sparse access point scenario with a room and local deviation of 8 dB and 0 dB. In this scenario, the absolute values of the $DIFF_{room}^{i,k}$ to be learned are on average 6.1 dB. The average absolute difference between the true and learned values, after each training iteration, are: 1.5 dB, 1 dB, 0.7 dB, 0.6 dB, and stay at 0.5 dB from the fifth training iteration onwards. A local deviation of 0 dB means that all $DIFF_{local}^{i,j}$ values are zero, which makes it easier to learn the correct $DIFF_{room}^{i,k}$ values but is not a realistic scenario as shown in Section 4.1. The proposed unsupervised learning technique always improves the location accuracy, except for low values of the room deviation in combination with high values of the local deviation. This is to be expected as the $DIFF_{local}^{i,j}$ values are high, which act as an additional source of noise but the $DIFF_{room}^{i,k}$ values are near zero, which is also not a realistic scenario as shown in Section 4.1. The nearest values to the experimentally derived room and local deviation from Section 4.1 for the WHIPP path loss model would lead to an improvement of 40.7%, 40.1%, and 40.3% in median accuracy, for the three access point scenario’s, respectively. Note that these simulations do not take into account the influence of additional noise.

#### 5.2.2. Influence of Additional Noise

**Figure 6:**
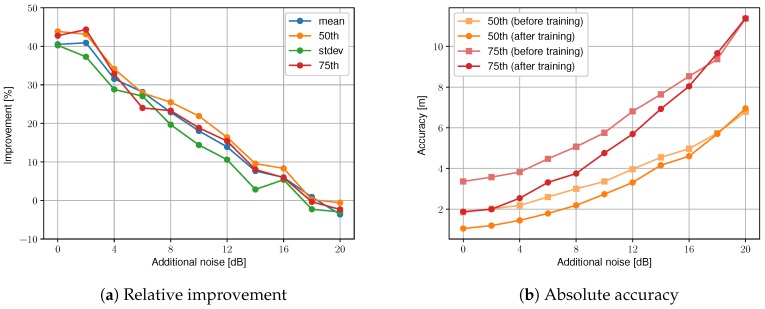
Accuracy before and after unsupervised learning for a varying level of additional noise with the normal access point configuration.

A more realistic scenario is to include the additional noise caused by, e.g., temporal fading or human body shadowing. Figure 6a shows the relative improvement in mean, 50th, standard deviation, and 75th percentile of the accuracy for increasing noise levels. Figure 6b shows the absolute 50th and 75th percentile values of the accuracy before and after training. The unsupervised learning phase is the same as in the previous section: each noise level is trained with 1 h of unlabeled training data, for a maximum of 10 training iterations, and is validated on 1000 uniformly spread locations. The access point configuration is set to normal (15 access points) and the room and local deviation are set to 5.7 dB and 3.7 dB, which are the experimentally derived values from Section 4.1 for the WHIPP path loss model. The improvement in mean, median, standard deviation, and 75th percentile value of the accuracy show a very similar trend. The median location accuracy improvement starts at 43.8% (from 1.85 m to 1.04 m) without additional noise (0 dB) and decreases roughly linear. The accuracy of the reconstructed training data needs to be at least accurate on room level, otherwise the radio map’s reference values are affected by measurements from a neighboring room, which will worsen the accuracy in the next iteration. The proposed unsupervised learning technique can improve the radio map and localization accuracy up to an additional noise of 16 dB, from then onwards the improvements are negative.

## 6. Experimental Validation

**Figure 7:**
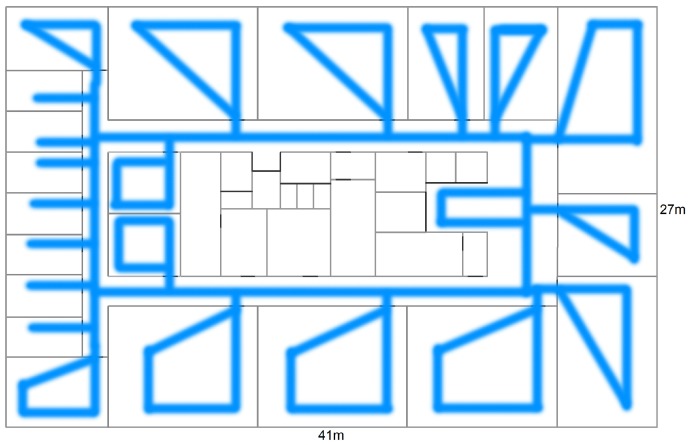
Floor plan with rough indication of the training data.

The test data for the experimental validation are the 200 static locations, uniformly spread over a whole floor in an office building (Section 4.1, Figure 2). The goal is to improve the quality of a model-based radio map and the location accuracy. The training data consists of a random walk of 15 min (or 900 location estimates) along the corridor, kitchen, offices, and meeting rooms, and is roughly indicated with blue lines in Figure 7.

Note that during the random walk the mobile node is hand-held instead of mounted on a tripod which causes additional deviation, as was previously mentioned. The exact positions are unknown and not needed for the learning phase, hence unsupervised, but are indicated to give an idea of the covered area. For obvious reasons, only the rooms were the random walks pass by, can be learned. Therefore, most areas were covered except for the server room, elevators, bathrooms, stairwells, and storage rooms in the center. Every second a location update is generated, the average RSS values of the packets received within this second are used as input for the route mapping filter. Next, the estimated positions serve as input to the radio map update step.

**Figure 8:**
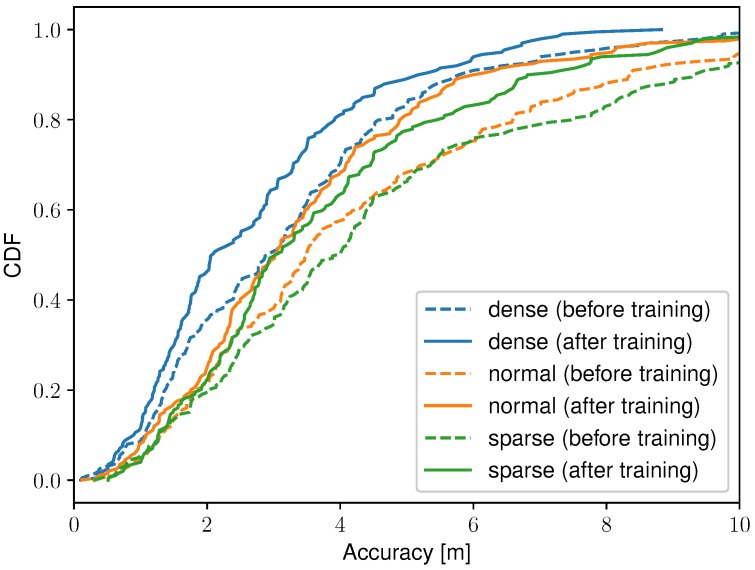
Cumulative distribution function of the localization accuracy before (dashed line) and after training (solid line), for the three access point configurations and the WHIPP path loss model.

Figure 4 shows the $DIFF_{room}^{i,k}$ values before and after the radio map optimization for one access point in the dense scenario. The colors are more grayish compared to the initial situation, which indicates that the proposed technique learns the correct values and improves the radio map. For this scenario, the average absolute difference between the experimentally derived and learned $DIFF_{room}^{i,k}$ values decreases from 7.4 dB to 4.0 dB for the free-space model, from 5.7 dB to 3.7 dB for the TGN model, and from 4.7 dB to 2.9 dB for the WHIPP model. Note that in rooms where no training data passes, no values can be learned, i.e., their color remains the same. The accuracies and relative improvement, before and after training, are summarized for all access point configurations and path loss models in Table 2. Figure 8 shows the cumulative distribution function (CDF) of the localization accuracy before and after training, for the three access point configurations and the WHIPP path loss model. These accuracies are based on the 200 static locations estimated with the initial and optimized radio map, and the weighted least squares algorithm (Equation (17)). The route mapping filter is only used to reconstruct the unlabeled training data.

The initial accuracies before training are similar for the three path loss models, the largest difference in mean accuracy is 0.69 m (5.04 m and 4.35 m between the free-space and TGn model for the sparse access point scenario). The standard deviation of the accuracy is always best with the WHIPP model because the free-space and TGn model show larger positioning outliers on locations were there is a lot of additional path loss. The latter is caused by the concrete walls, which are better modeled in the radio map of the WHIPP model. The scenario with the dense access point configuration and WHIPP path loss model has the highest improvement; the median accuracy improves from 2.90 m to 2.07 m (28.6%). For this scenario, the median accuracy starts at 2.90 m and is consecutively: 2.40 m, 2.33 m, 2.22 m, 2.15 m, and finally 2.07 m after the fifth training iteration. The learned $DIFF_{room}^{i,k}$ values, and hence the median location accuracy, remained the same in the sixth training iteration, which ended the optimization process. The largest relative improvement always occurs in the first training iteration and the learned values, and hence, the location accuracy, stagnate after a maximum of six iterations, for all scenarios. There is only one substantial negative improvement, the 75th percentile accuracy for the free-space model in the dense access point configuration, decreases from 3.61 m to 3.98 m or a degradation of 10.4%. This happens when the training data’s estimated trajectory deviates too much from the ground truth locations, which causes the fingerprint database to learn RSS values measured in another room.

The highest accuracy, for all metrics and access point configurations, is achieved by the optimized WHIPP model, despite that in some scenarios the initial accuracy with the free-space or TGn model was better before training. The initial accuracy for those models are adequate due to the large number of line-of-sight connections with the dense access point configuration, resulting in stronger signals, which have a higher weight in the static location algorithm (Section 5). Furthermore, the improvement in their learning phase is limited because larger outliers occur with the free-space model and TGn model, e.g., if training measurements get assigned to a wrong room then this room attracts similar measurements in the next training iteration. Averaged over all access point configurations, the WHIPP model shows an improvement in mean, standard deviation, median, and 75th percentile accuracy of 18.6%, 20.7%, 21.9%, and 21.3%, respectively. This is similar to the simulation with the experimentally derived room and local deviation, and an additional noise level of 8 dB (Figure 6). The IEEE 802.11 TGn model is only slightly worse than the WHIPP model which makes it a valuable alternative if the access point locations are known but further information about the building’s layout is limited. The free-space model can result in adequate results as long as the access point configuration is not sparse but since the implementation effort is the same as with the TGn model, the latter is preferred.

## 7. Conclusions

This work presents an unsupervised learning technique to construct and optimize model-based radio maps or fingerprint databases for indoor positioning systems, e.g., to make the radio map more accurate or to automatically cope with changes in an office layout. The proposed technique does not rely on time-consuming measurement campaigns, device calibrations, or additional inertial measurement units, that are power consuming. Instead, it uses an initial radio map based on a theoretical path loss model, unlabeled training data, a self-calibration method, and a route mapping filter. The premise of this work is that the differences between real measurements and reference values, derived from a model-based radio map, tend to be correlated per room and access point. Three theoretical path loss models are considered: the free-space model, the IEEE 802.11 TGn model, and a model that takes into account wall and interactions losses (WHIPP). Simulations showed that the discrepancies between reference fingerprints and real measurements could be learned in various scenarios, based on the random walks that a typical person does. This results in reference fingerprints that match the real measurements more closely, and hence, will lead to better radio maps and location accuracies. An experimental validation on a testbed in an office building in Ghent, Belgium, confirmed the simulations. The highest relative improvement is 28.6%, the median accuracy with the WHIPP path loss model improved from 2.90 m to 2.07 m after unsupervised learning with only 15 min of unlabeled training data. Furthermore, it is shown that the IEEE 802.11 TGn model is a valuable alternative if the information about the building’s layout is limited. Future work should include test and training data with multiple, simultaneously active users, covering multiple floors, other buildings, influence of access point location uncertainty, and ability to recover from physical changes in the environment.

## Figures and Tables

**Table 1:** Experimentally measured differences and deviations compared to the theoretical path loss models after self-calibration.

Path Loss Model	Difference [dB]			Deviation [dB]		
	min	max	avg	Overall	Room	Local
Free-space	−30.3	19.7	−0.4	10.9	9.7	3.5
IEEE 802.11 TGn	−37.5	19.1	−0.7	9.6	8.8	3.5
WHIPP	−22.0	24.0	0.8	7.6	5.7	3.7

**Table 2:** Accuracy of experimental validation test set per access point (AP) configuration and path loss (PL) model. The first and second value are the accuracy before and after training and the third value is the relative improvement.

#APs	PL Model	Accuracy [m]			
		*μ*	*σ*	50th	75th
9 (sparse configuration)	Free-space	5.04 → 5.11 (−1.3%)	3.93 → 3.98 (−1.3%)	4.12 → 3.93 (4.8%)	6.50 → 6.35 (2.2%)
	TGn	4.35 → 4.23 (2.9%)	3.73 → 3.72 (0.3%)	3.14 → 3.07 (2.1%)	5.72 → 5.13 (10.4%)
	WHIPP	4.66 → 3.77 (19.0%)	3.24 → 2.49 (23.3%)	3.94 → 3.03 (23.3%)	5.97 → 4.83 (19.1%)
15 (normal configuration)	Free-space	4.28 → 3.97 (7.4%)	3.43 → 2.88 (16.1%)	3.49 → 3.40 (2.4%)	5.03 → 4.99 (0.8%)
	TGn	4.22 → 3.96 (6.0%)	3.48 → 3.18 (8.7%)	3.42 → 3.31 (3.2%)	5.42 → 4.71 (13.0%)
	WHIPP	4.33 → 3.50 (19.1%)	2.98 → 2.38 (20.0%)	3.50 → 3.02 (13.7%)	6.03 → 4.44 (26.4%)
35 (dense configuration)	Free-space	3.13 → 3.22 (−2.9%)	3.09 → 2.62 (15.5%)	2.40 → 2.30 (4.1%)	3.61 → 3.98 (−10.4%)
	TGn	3.65 → 2.92 (20.1%)	3.18 → 2.10 (33.9%)	2.75 → 2.43 (11.7%)	4.51 → 3.65 (19.1%)
	WHIPP	3.23 → 2.66 (17.6%)	2.14 → 1.74 (18.7%)	2.90 → 2.07 (28.6%)	4.31 → 3.52 (18.4%)
